# Draft genome sequences of six bacterial strains degrading the biodegradable plastic polyhydroxybutyrate (PHB)

**DOI:** 10.1128/mra.00105-25

**Published:** 2025-03-27

**Authors:** Rino Isshiki, Kyohei Kuroda, Riho Tokizawa, Chisato Shiiba, Shodai Hino, Naoko Yamano, Hideyuki Tamaki, Atsuyoshi Nakayama, Takashi Narihiro, Kyosuke Yamamoto

**Affiliations:** 1 Bioproduction Research Institute, National Institute of Advanced Industrial Science and Technology (AIST) 74009 , Sapporo, Hokkaido, Japan; 2 Bioproduction Research Institute, National Institute of Advanced Industrial Science and Technology (AIST), Tsukuba, Ibaraki, Japan; 3 Biomedical Research Institute, National Institute of Advanced Industrial Science and Technology (AIST) 73773, Ikeda, Osaka, Japan; University of Southern California, Los Angeles, California, USA

**Keywords:** draft genomes, *Sessilibacter*, *Roseibium*, biodegradable plastic, polyhydroxybutyrate (PHB), marine biodegradation

## Abstract

The draft genome sequences of six strains of bacteria degrading polyhydroxybutyrate (PHB), a biodegradable plastic, have been sequenced. These strains were isolated from marine biodegradation test samples of PHB. The genome sizes range from 4.5 to 6.6 Mb, and they are phylogenetically distinct from known PHB-degrading bacteria.

## ANNOUNCEMENT

Polyhydroxybutyrate (PHB) is known as a raw material for biodegradable plastics, which are being studied as an alternative to petroleum-derived plastics from environmental perspectives (1). To date, PHB-degrading bacteria have been found in diverse environments (2–7), while the culture-independent metagenomic analysis suggests the existence of many uncultivated PHB-degrading bacteria (8).

**Fig 1:**
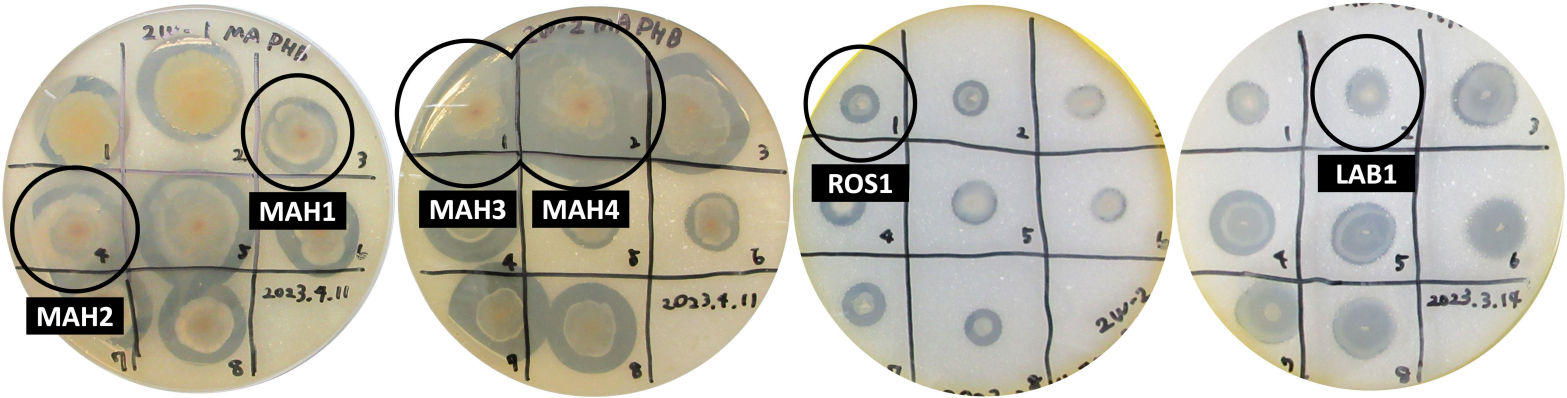
Clear zone formed by the degradation of PHB.

Here, the PHB-degrading bacteria were isolated from the biodegradability test samples using seawater (9) as the source, and their genomes were sequenced. The seawater was collected from the sea surface at a coastal area in Japan (34°37′03.2″N 135°25′42.1″E). Then, 30 mg of PHB powder (Merck, Germany) and 200 mL of seawater were added to testing bottles without adding any sources of nutrients and incubated at 27°C under stirring. The PHB degradation was evaluated from determination of O_2_ consumption using laboratory-scale BOD testers (BOD200F, TAITEC, Japan/OxiTop IDS, WTW, Germany) (10). Next, the culture was spread on a double-layered 1.5% agar plate containing artificial seawater DAIGO (SHIOTANI M.S., Japan) and yeast extract or marine agar in the bottom layer and 1.2% PHB fine powder in the top layer. After 2 weeks of incubation at 25°C, colonies forming a clear zone, which indicated PHB degradation activity, were selectively isolated (Fig. 1). The isolation process was repeated three times for purification. Cells for genome analysis were obtained by centrifuging and pelleting liquid culture samples or collecting colonies on agar plates cultured at 25°C in DAIGO containing yeast extract.

The genomic DNA was extracted using a NucleoSpin Microbial DNA kit (Macherey-Nagel, Germany) following the manufacturer’s instructions. Whole-genome shotgun sequencing was conducted using the Illumina NovaSeq6000 platform (Illumina Inc., USA) at the NovoGene Co., Ltd. (Beijing, China). A short-read library was prepared using DNA fragmented by Covaris ultrasonic shearing system (Covaris Inc., USA) and NEBNext Ultra II DNA Library Prep Kit for Illumina (New England Biolabs Inc., USA). Illumina paired-end sequences (2 × 150 bp) were determined using a NovaSeq 6000 S4 Reagent Kit v1.5 (Illumina). Read QC and adapter trimming were performed using Fastp v0.23.1 (11). The genomes were assembled using SPAdes v3.15.3 (12). Structural annotation of the genomes was performed using the DDBJ Fast Annotation and Submission Tool (DFAST) pipeline version 1.2.20 (13) with the following programs: GAPannotator version 1.0 (14) for assembly gap, MetaGeneAnnotator version 2008/08/19 (15) for CDS, CRT version 1.2(16) for CRISPR, Aragorn version 1.2.38 (17) for tRNA, and Barrnap version 0.8 (18) for rRNA. Default parameters were used for all software unless otherwise specified.

The six genomes ranged in length from 4.49 to 6.65 Mbp and in GC content from 42.3% to 59.0% (Table 1). The average nucleotide identity (ANI)-based phylogeny calculated by the DFAST_QC pipeline version 0.5.7 (19) indicated that four strains were closely related to the genus *Sessilibacter*, and the other two strains were closely related to *Roseibium* with an ANI of 78% and 88%, respectively. The PHB depolymerase gene [EC 3.1.1.75] was annotated on all six genomes. As any PHB-degrading strains belonging to these two genera have not been reported, further investigation is needed to clarify PHB degradation mechanisms and ecological role in the marine ecosystem.

**TABLE 1:** Sequencing data and genome characteristics for six strains of PHB-degrading bacteria

Strain	*Sessilibacter* sp. MAH1	*Sessilibacter* sp. MAH2	*Sessilibacter* sp. MAH3	*Sessilibacter* sp. MAH4	*Roseibium* sp. LAB1	*Roseibium* sp. ROS1
Total contig length (bp)	4,539,058	4,542,034	4,494,785	4,494,868	6,187,225	6,646,216
Number of contigs	38	41	67	73	18	35
Longest contigs (bp)	442,042	411,077	405,843	405,843	2,164,360	1,792,396
Sequencing coverage (×)	450	324	361	320	303	275
N50 (bp)	208,135	184,078	139,811	118,930	1,719,597	1,781,916
Gap ratio (%)	0.019299	0.019198	0.010768	0.01079	0.001568	0.004409
GC content (%)	42.3	42.3	42.7	42.7	59.3	59
Number of CDSs	3,712	3,711	3,685	3,683	5,703	6,074
Average Protein Length (aa)	323.2	323.2	323.7	323.9	317.5	318.1
Coding ratio (%)	79.3	79.2	79.6	79.6	87.8	87.2
Number of rRNAs	3	3	3	3	3	3
Number of tRNAs	38	38	38	38	48	51
Number of CRISPRs	2	2	2	3	0	0
Completeness (%)	100	100	100	100	100	100
Contamination (%)	0	0	0	0	0	0
ANI-based taxonomy (FastANI)	Sessilibacter corallicola C21	Sessilibacter corallicola C21	Sessilibacter corallicola C21	Sessilibacter corallicola C21	Roseibium aggregatum IAM 12614	Roseibium aggregatum IAM 12614
ANI (%)	77.6086	77.7527	77.9442	77.9386	87.8181	88.0352
SRA accession no.	DRR635727	DRR635728	DRR635729	DRR635730	DRR635731	DRR635732
Assembly accession no.	BAAGIC010000000	BAAGID010000000	BAAGIE010000000	BAAGIF010000000	BAAGIB010000000	BAAGIG010000000
